# Evaluation of concentration procedures, sample pre-treatment, and storage condition for the detection of SARS-CoV-2 in wastewater

**DOI:** 10.1007/s11356-023-29696-y

**Published:** 2023-09-21

**Authors:** Francesca Cutrupi, Michele Rossi, Maria Cadonna, Elisa Poznanski, Serena Manara, Mattia Postinghel, Giulia Palumbi, Marta Bellisomi, Elena Nicosia, Giorgia Allaria, Lorenzo Dondero, Carolina Veneri, Pamela Mancini, Giusy Bonanno Ferraro, Giuseppina Rosa, Elisabetta Suffredini, Paola Foladori, Elena Grasselli

**Affiliations:** 1 Department of Civil, Environmental and Mechanical Engineering, University of Trento, via Mesiano 77, 38123 Trento, Italy; 2 Department of Biosciences, University of Milano, Via Celoria 26, 20134 Milano, Italy; 3 ADEP, Agenzia per la Depurazione (Wastewater Treatment Agency), Autonomous Province of Trento, via Gilli 3, 38121 Trento, Italy; 4 Via della Mendola 13, 39100, Bolzano, Italy; 5 Department of Cellular Computational and Integrative Biology-CIBIO, Via Sommarive 9, 38123 Trento, Italy; 6 ARPAL Virology and enviromental biotecnological laboratory, Genova, Liguria Italy; 7 Department of Health and Social Services, Liguria Region Administration, Piazza della Vittoria 119, 16121 Genova, Italy; 8 Department of Earth Sciences of the Environment and Life, University of Genova, Corso Europa 26, 16132 Genova, Italy; 9 Department of Environment and Health, Istituto Superiore di Sanità, Rome, Italy; 10 Department of Food Safety, Nutrition and Veterinary Public Health, Istituto Superiore di Sanità, Rome, Italy

**Keywords:** SARS-CoV-2, Wastewater, Wastewater-based epidemiology, Concentration methods, Storage temperature, Pasteurization

## Abstract

Crucial information on the pandemic’s spread has been gathered by monitoring the trend of SARS-CoV-2 in wastewater. This surveillance has highlighted that the initial concentration is a critical step of the analytical procedure due to the low viral titer that may be present in this matrix. This paper presents the results of the evaluation of two different wastewater concentration protocols to determine the most efficient and cost-effective. The two methods tested were the following: (a) a biphasic separation system with PEG-dextran and (b) a PEG/NaCl precipitation protocol. Other aspects of the detection method were also investigated including the influence of storage temperature on virus recovery and the heat treatment of pasteurization, which aims to make samples safer for operators and the environment. The PEG/NaCl precipitation method was found to perform better than the biphasic separation system, allowing for more sensitive identification of the presence of the virus and the detection of a higher viral titer than that identified with the biphasic separation in all results. Storage of the samples at 4.3±0.2°C for up to 3 weeks did not adversely affect the virus titer and the pasteurization pre-treatment increases operator safety and maintains the identification of the viral concentration.

**Graphical abstract:**

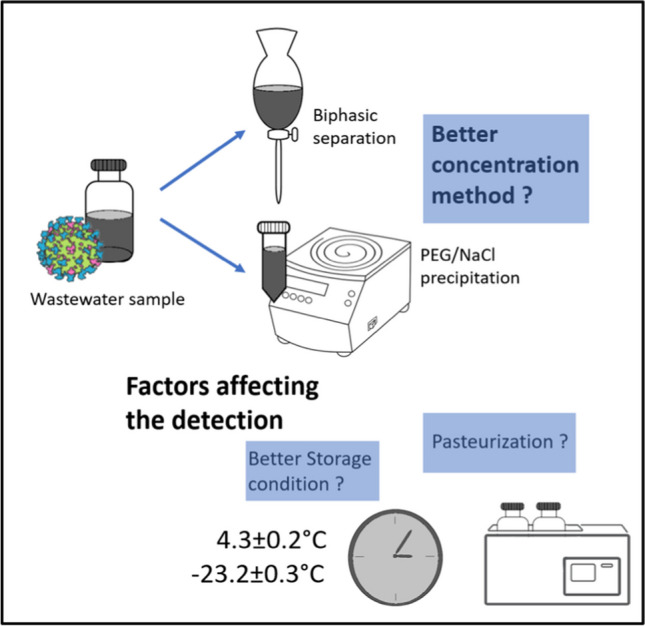

**Supplementary Information:**

The online version contains supplementary material available at 10.1007/s11356-023-29696-y.

## Introduction

Wastewater-based epidemiology (WBE) implies continuous monitoring by measuring biological or chemical indicators in sewage to provide information on a community’s collective health status or habits. Compared to other surveillance approaches, WBE is a cost- and time-efficient tool for detecting pathogens and quantifying community prevalence. WBE enables us to vastly increase the number of individuals under surveillance compared to clinical data, as it leverages the wastewater from an entire urban sector collected through the sewer network. Comparing these approaches to surveillance from clinical data, the WBE presents a comprehensive view of the manifestations of pathogenic infection because it collects data not only from symptomatic but also from pre-symptomatic and asymptomatic individuals (He et al. 2020; Wang et al. 2020). However, it does not allow the punctual identification of these individuals. As a result, wastewater can be considered an important source of information for pandemic surveillance (Mallapaty 2020; Randazzo et al. 2020). Moreover, the versatility of this monitoring tool makes it applicable for two key objectives: to provide early warning of disease outbreaks and to assess the effectiveness of public health interventions, as the immunization campaigns, already documented in studies on viruses such as norovirus, hepatitis A virus, and poliovirus (Huang et al. 2022; Zuckerman et al. 2022).

Coronavirus disease 2019 (COVID-19), the disease caused by Severe Acute Respiratory Syndrome Coronavirus 2 (SARS-CoV-2), was declared a pandemic by the World Health Organization (WHO) on March 11, 2020 (WHO 2020). During this unprecedented challenge, significant efforts have been made to prevent and overcome the progression of the pandemic, despite the uncertainty regarding its duration and trajectory. Crucial information on the pandemic’s spread has been gathered by monitoring the trend of SARS-CoV-2 in wastewater. To do this, the virus concentration values in the wastewater were compared with the data on the number of infected from monitoring the nasopharyngeal swabs of the population (Cutrupi et al. 2022). By comparing these trends, it was possible to confirm the early warning signal for outbreaks, showing the predictive nature of WBE and the effectiveness of public health interventions (Melvin et al. 2021), making this type of surveillance a valuable tool for the management of the COVID-19 pandemic.

In Italy, the SARI project (Epidemiological Surveillance for SARS-CoV-2 in urban sewage) coordinated by Istituto Superiore di Sanità (ISS) has been active since July 2020. The project established a national network with the cooperation of several entities, including Regions, Autonomous Provinces (A.P.), wastewater service providers, regional environmental protection agencies, local health authorities, zooprophylactic institutes, universities, and research institutions. Within this network, data and experimental approaches were shared to harmonize SARS-CoV-2 monitoring experimental approaches leading to greater consistency in the data produced by laboratories across all Regions/A.P.s of Italy (Ministero della Salute 2020).

As of May 4th, 2023, the WHO has declared the end of the pandemic emergency (WHO 2023a). While COVID-19 continues to be recognized as a global health threat, the WHO’s emergency committee made this decision based on several factors. They observed a decreasing trend in COVID-19 deaths, a decline in related hospital admissions and intensive care cases, and the presence of high levels of population immunity to SARS-CoV-2. As a result, COVID-19 is now considered an established and ongoing health matter, no longer constituting a public health emergency of international concern.

Considering this development, the WHO recommends that countries transition from emergency response mode to long-term management of COVID-19, alongside other infectious diseases. To aid countries in managing the virus on an ongoing basis, the WHO is establishing a review committee to develop long-term recommendations. Additionally, the fourth edition of its global strategic preparedness and response plan for COVID-19 has been published, covering collaborative surveillance, community protection, safe and scalable care, access to countermeasures, and emergency coordination (WHO 2023b).

These efforts reflect a shift in approach, moving from emergency response to sustainable management and preparedness for future challenges related to COVID-19 and other pathogens. The WHO’s continued focus on collaborative efforts and strategic planning aims to address the health implications of the virus while fostering resilience and effective response strategies on a global scale.

The end of the pandemic emergency offers the opportunity to draw valuable lessons from that difficult period. From the specific experience of SARS-CoV-2 monitoring, which can be seen as a case study, we can focus on implementing the WBE and its applications in the surveillance of other viruses and pathogens, starting from the same sampling effort and even from the same sample.

First, implementing the WBE approach presents several challenges. Regarding the reliability of the surveillance results, some aspects, such as the variable characteristics of the sewage network, the geographical basin, and the habits of the population, such as the fluctuation of residents due to the presence of tourists, have an essential weight and necessitate careful consideration (Jiang et al. 2023; Oloye et al. 2023).

Moreover, the integrity of genomic RNA is greatly challenged by the complexity of the wastewater and RNase present in the matrix (Philo et al. 2021). Also, these variables can affect the reliability and accuracy of the data obtained from wastewater surveillance. The RNA yield can vary depending on several factors, including the type of sampling (composite or instantaneous), sample pasteurization, storage time, and temperature.

About the temperature, it is important to maintain the cold chain during the transport and storage of samples to ensure that the integrity of the viral RNA is not compromised. Samples should ideally be analyzed fresh within 24 h of receipt at the laboratory. However, during long-term surveillance, depending on the distance between the WWTPs and the laboratory or the workload, it may be necessary to store some samples frozen under −18°C or refrigerate at a temperature between +2 and +8°C until analysis. These temperatures are the storage temperatures commonly found in laboratories belonging to the SARI network.

Another aspect to be explored, linked to the possibility of identifying pathogens in wastewater, concerns the low viral titers in wastewater following the dilution of human excreta through the sewage system.

That said, many methods have been published in the literature for the detection of SARS-CoV-2 (inter alia Ahmed et al. 2020; Randazzo et al. 2020, Wu et al. 2020); however, given the high variability of the source material, it is crucial to test the validity of each method in the specific context of wastewater analysis.

Through this paper, we aim to share our experience in identifying a method for the detection of SARS-CoV-2, not only to document the need for an easily replicable method but also to share the insights of the Italian network in this regard with the hope that what was discovered for SARS-CoV-2 can be generalized to the detection of other viruses and pathogens, in the context of the WBE. Here we present the results of the comparative tests performed by some laboratories of the SARI network on two protocols for concentrating SARS-CoV-2 in wastewater: the biphasic separation system with PEG-dextran and the PEG/NaCl precipitation protocol and other aspects that may influence this analysis.

## Materials and methods

### Wastewater samples

A total of 283 samples of raw wastewater (24-h composite) were collected from two Italian Regions and one A.P. (Lazio, Liguria, and Trento Province), located in central and northern Italy, between October 2020 and April 2021. The samples were collected from 22 wastewater treatment plants (WWTPs) of different sizes, ranging from 17,500 to 1,100,000 Population Equivalents. The raw wastewater was collected, with refrigerated autosamplers, at the WWTPs inlet after sieving and degretting treatment (where coarse materials and sand are removed) but before the primary settling. Volumes of 250 mL were transported refrigerated to the laboratories for analysis.

### Comparison of concentration methods

The concentration measures and the recovery efficiency of the two concentration methods were compared to develop an efficient and cost-effective protocol that allows the analysis of many samples in a short time. These methods were the following:

- a biphasic separation system with PEG-dextran, adapted from the protocol of the WHO Guidelines of 2003 for the Environmental Surveillance of Poliovirus (WHO 2003) for the detection of enveloped viruses, as shown in the work of La Rosa et al. (2020). In this method, 250 mL of wastewater was centrifuged for 30 min at 1200 ×g and the solid fraction was separated. Then, 20 mL of 22% dextran, 143 mL of 29% PEG6000, and 17 mL of 5N NaCl were added to the supernatant. After vigorous mixing, the solution was transferred to a separatory funnel and left to stand overnight at 4°C. Subsequently, the bottom layer and the interphase were added, drop by drop, to the solid fraction of the initial centrifugation. During this step, 8-10 mL of the solution was recovered; subsequently, chloroform was added at a ratio of 1:4 v/v. After a second centrifugation at 1000 ×g for 10 min, the supernatant was collected and divided into 2 aliquots, one for subsequent extraction and the other for further study.
- a PEG/NaCl precipitation protocol, modified by Wu et al. (2020), widely recognized in the industry. In this method, 50 mL of the matrix was first centrifuged at 4500 ×g for 30 min at 4°C to remove large particles such as debris. From the supernatant, 40 mL was added to 4 g of 8% PEG8000 and 0.9 g of 0.3 M NaCl, stirred for 15 min until the chemicals were completely dissolved, and then centrifuged at 12000 ×g at 4°C for 2 h. Subsequently, the liquid fraction was discharged, and the pellet, often invisible, was resuspended with 2 ml of lysis buffer containing guanidine thiocyanate (bioMerieux), as a first step of the nucleic acid extraction procedure.

### Nucleic acid extraction

Viral RNA was extracted using automated and semi-automatic extraction platforms such as NucliSens® miniMAG™ and eGENE-UP® (bioMérieux, Marcy l’Etoile, France). After adding the lysis buffer, the samples were incubated for 20 min at room temperature. Then, 50 μL of magnetic silica beads were added to the sample and left for 10 min at room temperature to allow the RNA adhesion to the beads. The extraction system then went through a series of contaminants removal steps to clean the beads, and the nucleic acids were eluted to a final volume of 100 μL. Before molecular testing, the extracted nucleic acids were further purified using the OneStep PCR Inhibitor Removal Kit (Zymo Research, CA, USA) to reduce the concentration of potentially RT-qPCR-inhibiting substances.

### RT-qPCR analyses

The extracted RNAs were tested for the presence of SARS-CoV-2 and were also quantified using a one-step quantitative real-time PCR (RT-qPCR) assay (Table 1) targeting the ORF-1b (nsp14) region, described in La Rosa et al. (2021). Additional tests performed using the N1 nucleocapsid gene as target, as indicated by Lu et al. (2020), were described in Supplementary materials. A quantitation cycle (*Cq*) cut-off of 40 (*Cq* < 40) was applied for positive results.

**Table 1:** Primers and probes used in the study; nucleotide numbering based on SARS-CoV-2 (accession no. NC_045512)

Primer name	Nucleotide sequence (5′-3′)	Genome location	Reference
2297-CoV-2-F	ACATGGCTTTGAGTTGACATCT	18600–18621	La Rosa et al. 2021
2298-CoV-2-R	AGCAGTGGAAAAGCATGTGG	18680–18699	
2299-CoV-2-P	FAM-CATAGACAACAGGTGCGCTC-MGBEQ	18649–18668	

RT-qPCR assays were performed using the AgPath-ID ™ (Applied Biosystem-ThermoFisher) one-step RT-PCR reagent kit.

For the ORF-1b assay, each 25 μL reaction contained 250 nM of 2299-CoV-2-P, 500 nM of 2297-CoV-2-F, and 900 nM of 2298-CoV-2-R, 1 μL of 25 × RT-PCR Enzyme, 12.5 μL of 2 × RT-PCR Buffer, and 5 μl of nucleic acid extract. The thermocycling conditions consisted of 30 min at 50°C for reverse transcription, 10 min at 95°C for RT inactivation, and 45 cycles of 15 s at 95°C and 45 s at 60°C for amplification.

The RT-qPCR reactions were run on the Applied Biosystems™ 7500 (ThermoFisher Scientific) and the CFX96 Touch Real-Time PCR Detection System (Biorad) instrument. Each sample was run in duplicate, and the threshold was set in the middle of the exponential amplification phase in the log view. The concentration of SARS-CoV-2 was expressed in genome units per microliter (GU/μL). The dsDNA ORF-1b standard was provided by the Istituto Superiore di Sanità (Italy). The qualifications were considered acceptable if the standard curves had a slope close to −3.32 (between −3.1 and −3.6) and a regression coefficient *R*^2^≥0.98.

### Virus spikes, evaluation of recovery, and inhibition

To evaluate the protocol’s efficiency in concentrating and extracting SARS-CoV-2 RNA from wastewater and to validate the effectiveness of laboratory procedures, surrogate viruses, called process control viruses (PCVs), were spiked into the samples. In this work, the PCV used was Mengovirus (MgV), widely recognized and utilized as a reliable PCV in numerous virology studies and highly resilient under laboratory conditions. An amount of 100 μL of PCV was added to the samples, and the final RNA concentration was calculated using specific RT-PCR tests (more details in Supplementary Material A). The recovery efficiency was calculated using the equation:

$$\textrm{Recovery}\ \textrm{rate}\left(\%\right)={10}^{\left(\Delta Cq/\textrm{m}\right)}\times F\times 100$$

where ∆*Cq* = *Cq* of the spike PCV in the sample − *Cq* of the undiluted extracted PCV

*m* = slope of the PCV standard curve

*F* = fraction of the initial volume of the processed sample

In the absence of a general agreement on the acceptable recovery rate for SARS-CoV-2 in wastewater, we used the criterion of recovery rate >1% according to ISO 15216-1:2017, which concerns the quantification of viruses in complex food categories.

### Investigation of other factors affecting the detection of SARS-CoV-2

In addition to investigating the concentration phase, other aspects of the standardization of the detection protocol were also investigated, particularly those on which there was not scientific consensus. The factors analyzed all concerned the initial stages of the analysis.

#### Storage conditions

**Fig. 1:**
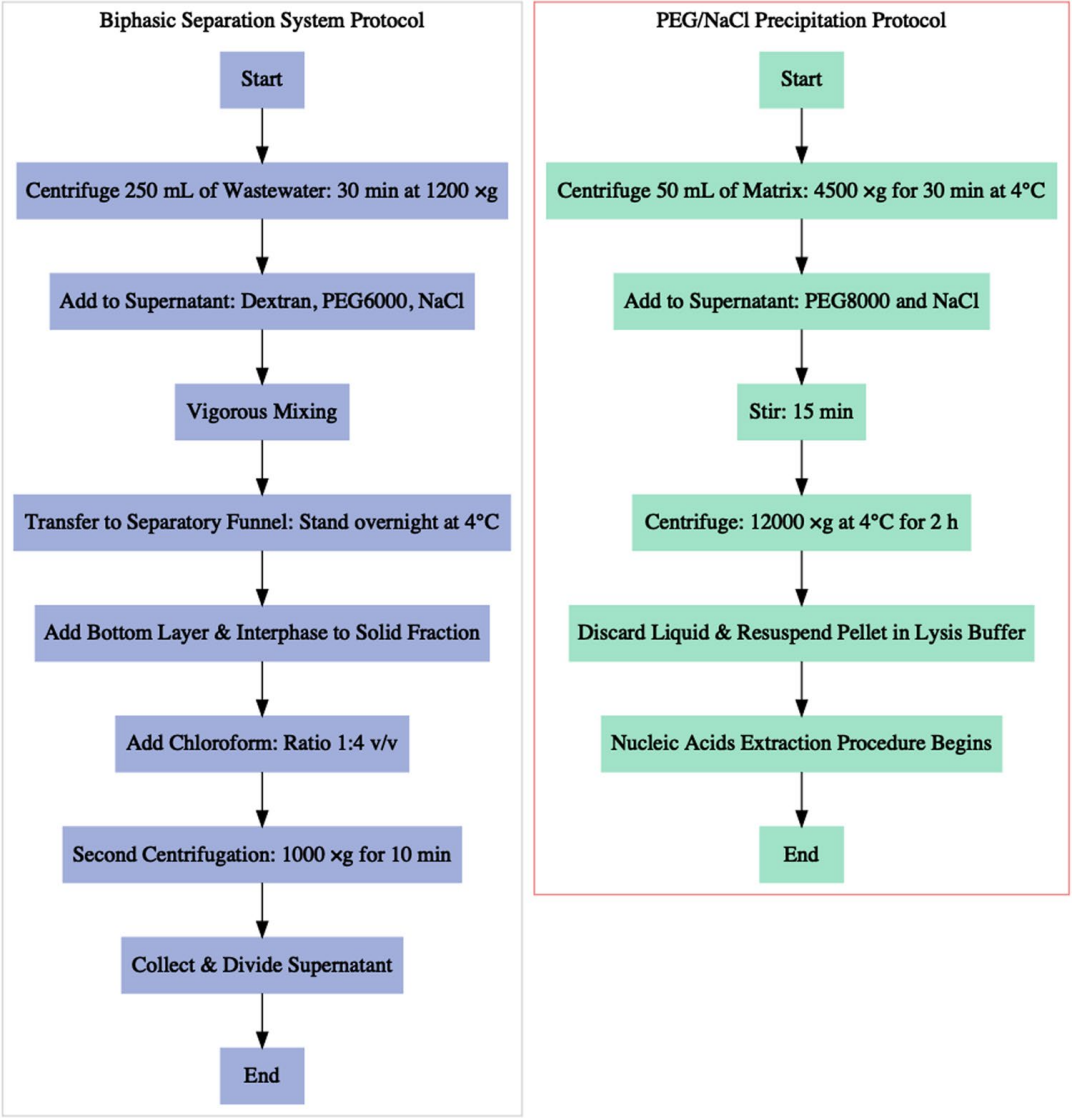
Flowchart of the compared protocols (biphasic separation system and PEG/NaCl precipitation protocol)

To study the influence of storage temperature on virus recovery, 19 aliquots of the same samples were stored in the condition of continued monitoring resulting in average temperatures of 4.3±0.2°C and -23.2±0.3°C and analyzed at days 0, 4, 5, 8, 12, 13, 15, 19, 20, and 22. The refrigerated aliquots were analyzed immediately after removal from the refrigerator, while the frozen ones underwent an initial thawing phase in the refrigerator lasting approximately 12 h. No samples were repeatedly frozen and thawed before testing. The analyses were performed with the PEG/NaCl precipitation protocol for the concentration phase and the RT-qPCR assay for the detection of the ORF-1b region (Fig. 1).

#### Pre-treatment: pasteurization

The pasteurization step in the protocol is intended to increase safety for the operators and the environment by reducing the risk of viral transmission. However, there have been conflicting opinions on the effect of pasteurization on the detection of SARS-CoV-2 RNA in wastewater. One concern is that the heat may destroy the viral material, making it difficult or impossible to detect. To evaluate the effect of pasteurization on SARS-CoV-2 RNA detection, 17 samples were collected from 3 WWTPs, aliquoted into duplicates, and analyzed with and without heat treatment (water bath at 56°C for 30 min), as reported in La Rosa et al. (2020). The samples were subsequently processed by comparing the two concentration methods and analyzed with the RT-qPCR ORF-1b assay.

### Statistical analysis

Data elaboration was performed using MS Excel, while the plots and the statistical analysis were carried out using R version 4.1.2. (The R Project for Statistical Computing 2021). Graphs were performed with ggplot 2 version 3.4.0 (ggplot2 2016) with some integration with introdataviz (Nordmann et al. 2022) to create visualizations to represent data.

## Results and discussion

### Comparison of concentration methods

The study analyzed a total of 283 samples, of which 147 were collected from 4 WWTPS in the Trentino Province, 106 from 14 WWTPs in the Region of Liguria, and 30 from 4 WWTPs in the Region of Lazio. The laboratory within each specific area conducted analysis on samples collected solely in the corresponding Region or Autonomous Province. The number of samples analyzed by each regional research group depended on the human availability, resource availability, and the temporal constraints of individual laboratories during a critical period such as that of the second wave of COVID-19 (winter 2020 - spring 2021).

**Fig. 2:**
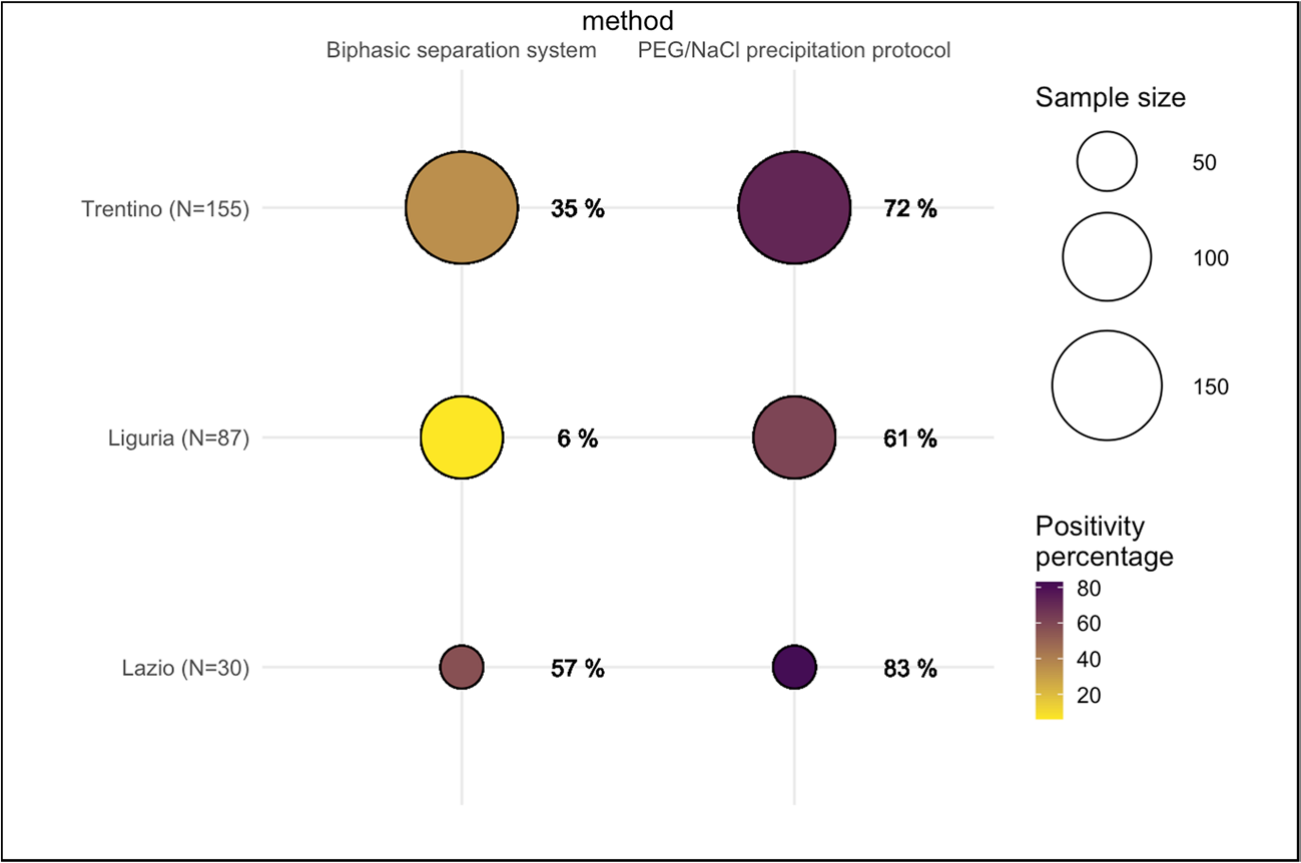
Positivity percentage obtained by the three research groups (Trentino, Liguria, and Lazio). The chart displays the sample size and the percentage of samples testing positive for the presence of SARS-CoV-2 using the biphasic separation system or the PEG/NaCl precipitation protocol

**Fig. 3:**
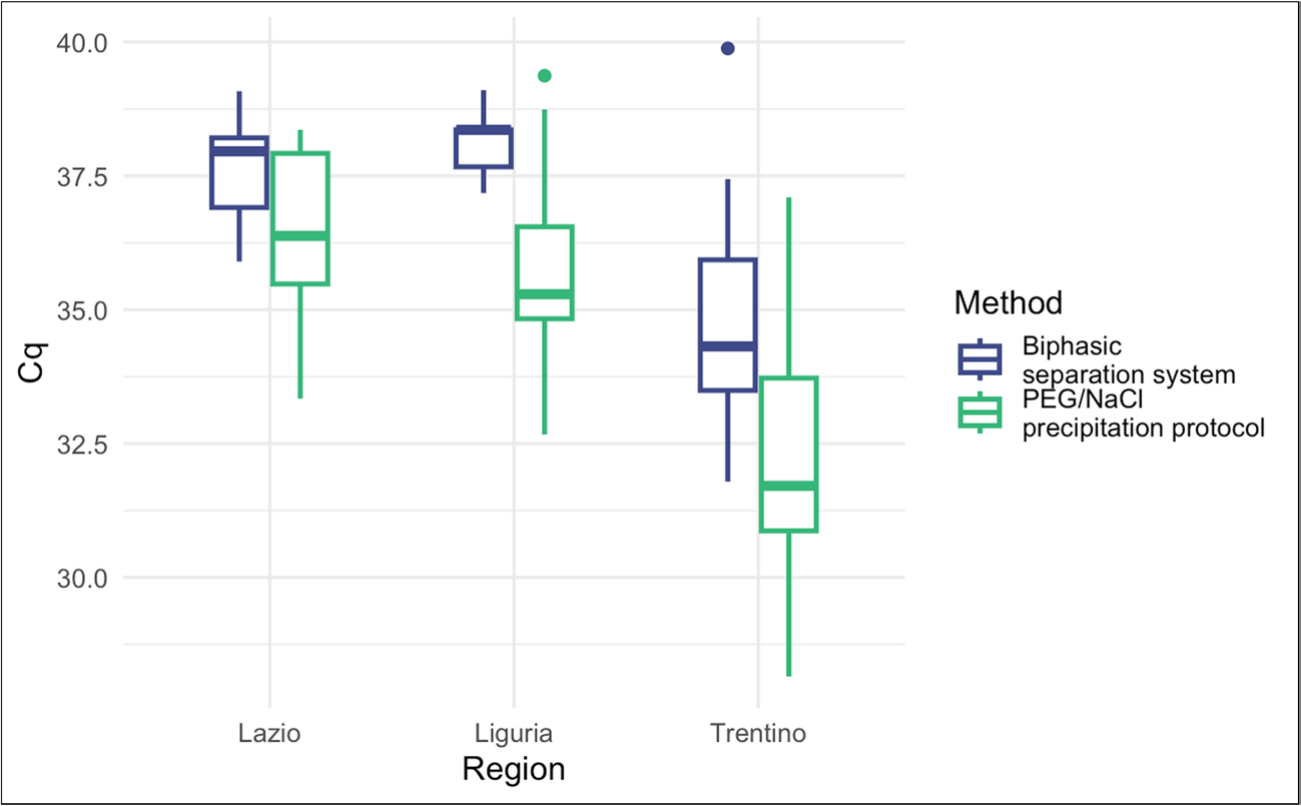
Comparison of the positive Cq results of the two protocols

Figure 2 presents the results of testing using the two methods indicated in the “Comparison of concentration methods” section. The viral concentrations were not normalized by population-equivalent, which influences the direct comparison of the results across different sites, as highlighted by LaTurner et al. (2021). For this reason, the results will be presented for single laboratories or molecular targets. However, common trends can be observed in the results of the single groups, as illustrated in Figs. 2 and 3.

The positivity percentage of the samples analyzed with the PEG/NaCl precipitation protocol was in the range 61–83% (Fig. 2). In contrast, the range of positivity for the biphasic separation method was lower, with the lowest percentage of 6% reported by the Liguria laboratory.

Figure 3 shows the *Cq* values of the positive samples tested with the two protocols in the three laboratories. The *Cq* results, obtained by RT-qPCR, are inversely proportional to the viral concentration detected in the samples. The PEG/NaCl precipitation method allowed a more sensitive identification of positivity in the presence of the virus and also the detection of a higher viral titre than that identified with the Biphasic separation in all the results.

The inter-laboratory differences observable in the *Cq* values presented in Fig. 3 may have various origins, including the variability in the concentration of the virus in the population served by the WWTPs and the natural fluctuations in the characteristics of wastewater (Islam et al. 2022; Juel et al. 2021). Nonetheless, the difference in results between the two methods is evident, as discussed below.

**Fig. 4:**
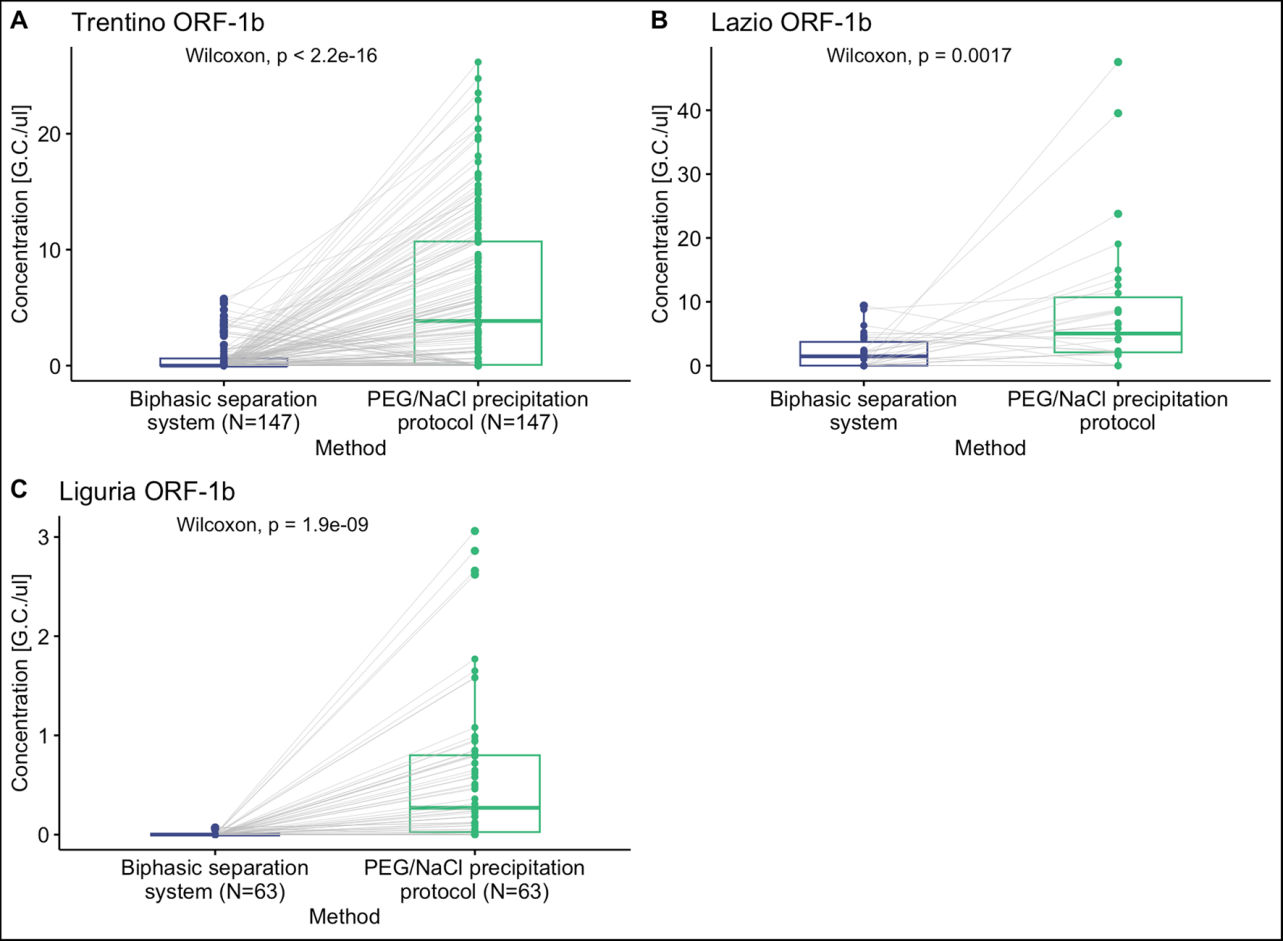
Comparison of SARS-CoV-2 concentration measured for each method (biphasic separation system and PEG/NaCl precipitation protocol) and groups: **A** Trentino, **B** Lazio, **C** Liguria for the molecular target ORF-1b. The boxplot, median values, and outliers are shown. The grey lines indicate the position of the same sample for the two methods compared. Outliers (highest 5%) were removed to allow better visualization

Figure 4 shows that higher SARS-CoV-2 concentrations were detected with the PEG/NaCl precipitation protocol compared to the biphasic separation method. The median concentrations are higher, as is the variability of the results, for the PEG/NaCl precipitation method compared to the biphasic protocol, which presents lower performances. Also, the junction lines of the same samples in the two methods confirm higher concentrations for the PEG/NaCl precipitation protocol.

The plots presented in Fig. 4A, B, and C, as well as the statistical test, were performed removing the highest 5% outliers obtained with the PEG/NaCl method and the corresponding Biphasic result. In all the sets of analyses, the Wilcoxon paired test confirmed the result difference between the two protocols.

To assess the interlaboratory variability and investigate the interaction effect between laboratory and method a two-way robust ANOVA, without outliers’ removal, was performed.

The results of the Wilcoxon paired test were confirmed as the method factor shows a significant effect on the concentration values (*p* < 0.001) and, similarly, the laboratory factor shows a significant effect (*p* < 0.001).

The interaction term is also significant (*p* < 0.001) indicating that the difference in results between the two methods varies depending on the laboratory where the tests were performed. However, given that PEG/NaCl precipitation is consistently superior in all laboratories, the intralaboratory variation does not change the overall conclusion that PEG/NaCl precipitation is more effective.

To date, the causes that lead to this difference in the results between the two concentration methods are not completely clear. This lack of clarity is also observable in other studies presenting a comparison of concentration protocols such as in LaTurner et al. (2021). It can be hypothesized that PEG plays an important role in increasing the concentration of the virus by precipitating it, and the removal of solids during the PEG/NaCl precipitation protocol may also contribute to the reduction of PCR inhibitory substances (Philo et al. 2021). However, it is important to note that the removal of solids on the efficacy of the virus concentration method is still unclear, as suggested by Pecson et al. (2021). Still, very good results in concentration measurements have also been obtained in other studies with methods using PEG precipitation which are shown to work better than filtration and adsorption methods (Dimitrakopoulos et al. 2022).

**Fig. 5:**
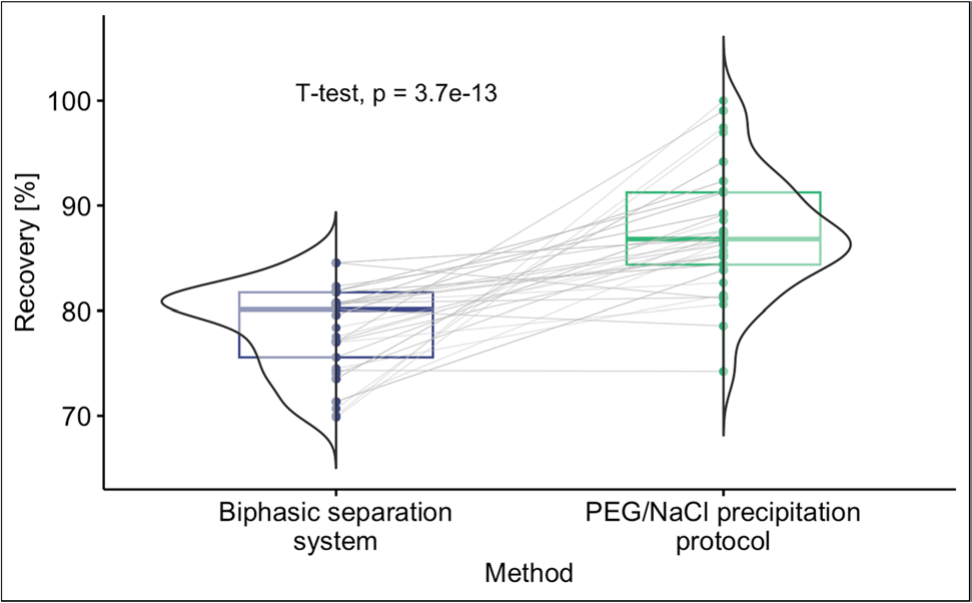
Percentage recovery of Mengovirus (MgV) for the two methods. In these plots, the values distributions of percentage recovery are represented by half violin plots showing the mono-modality of the data

To assess the quality of the analysis and the reliability and consistency of the results, it is necessary to use a surrogate virus as process control and evaluate its recovery rate (Juel et al. 2021). For both methods and molecular targets, as shown in Fig. 5, a wide range of MgV recovery was obtained due to the high sample variability, but always higher than the 1% minimum required by the ISO 15216-1:2017. A higher median value was observed for the PEG/NaCl precipitation analysis (around 88%) than the one of the biphasic separation protocol (80%). From the junction lines, most of the samples show a higher recovery with the PEG/NaCl precipitation system. The paired *t*-test, with *p* values shown in Fig. 5, confirms the different outcomes of the two methods. Several alternative PCVs, more or less similar to the target virus, could be chosen, and their recovery rates can vary widely based on the RNA concentration and extraction methods used (Juel et al. 2021; LaTurner et al. 2021). The lower recovery values in the biphasic separation system may be due to a lower detection efficiency of the target virus or a higher level of interference from PCR inhibitory substances (Philo et al. 2021).

### Investigation results of other factors affecting the detection of SARS-CoV-2

#### Storage conditions

**Fig. 6:**
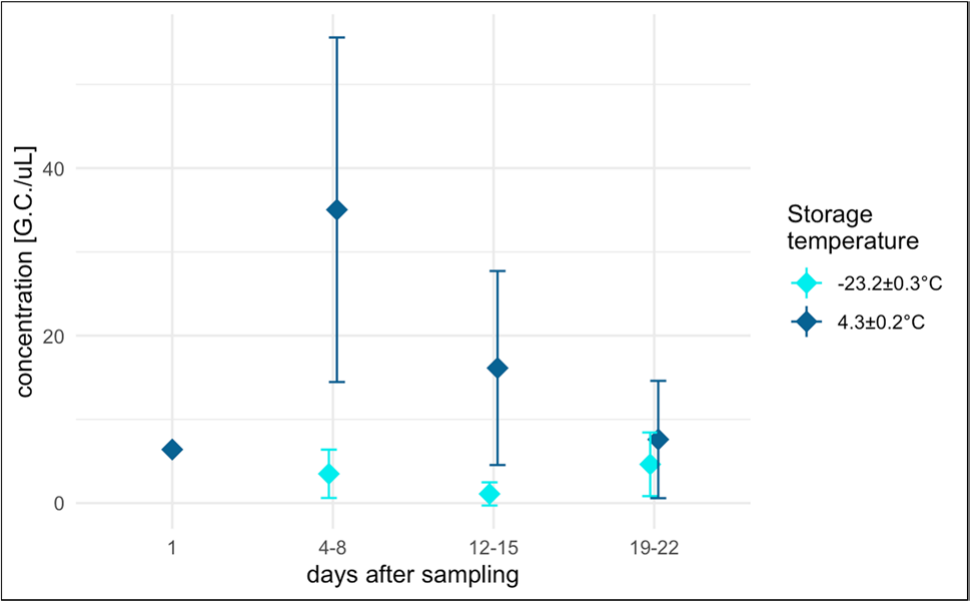
Concentration of SARS-CoV-2 in raw influent wastewater stored at 4.3±0.2°C and −23.2±0.3°C from 1 to 22 days. The range 4–8 comprises samples analyzed 4, 5, and 8 days after sampling, the 12–15 samples analyzed 12, 13, and 15 days after, and 19–22 samples from 19, 20, and 22 days after. Error bars represent the standard deviation between grouped samples

The results in Fig. 6 show the effect of storage temperature on viral detection and on the viral concentration of a sample over time. Aliquots of 250 ml of the same sample were stored at 4.3±0.2°C and −23.2±0.3°C, and were tested at different time points (1, 4, 5, 8, 12, 13, 15, 19, 20, and 22 days after sampling). The initial concentration at time 0 was 6.4 G.C/μL. After 2 weeks of storage at −23.2±0.3°C, the concentration decreased almost to the detection limit. Conversely, the concentration remained relatively stable at 4.3±0.2°C, however showing an increase in the first week. This peculiar result can be explained by hydrolysis and solubilization of the particulate matter, which can result in the release of viral material. However, similar results, which promote storage of samples at 4.3±0.2°C for the first 14 days, were published by Islam et al. (2022) and Mark et al. (2021). The slight decrease after storage at −23.2±0.3°C may be due to the freezing process. It is known that freeze-thaw cycles can lead to a degradation of nucleic acids through physical damage such as the formation of ice crystals that can alter the integrity of the capsid in viruses that present it or damage the strands of RNA and DNA (Röder et al. 2010; Kaya et al. 2022).

#### Pasteurization

**Fig. 7:**
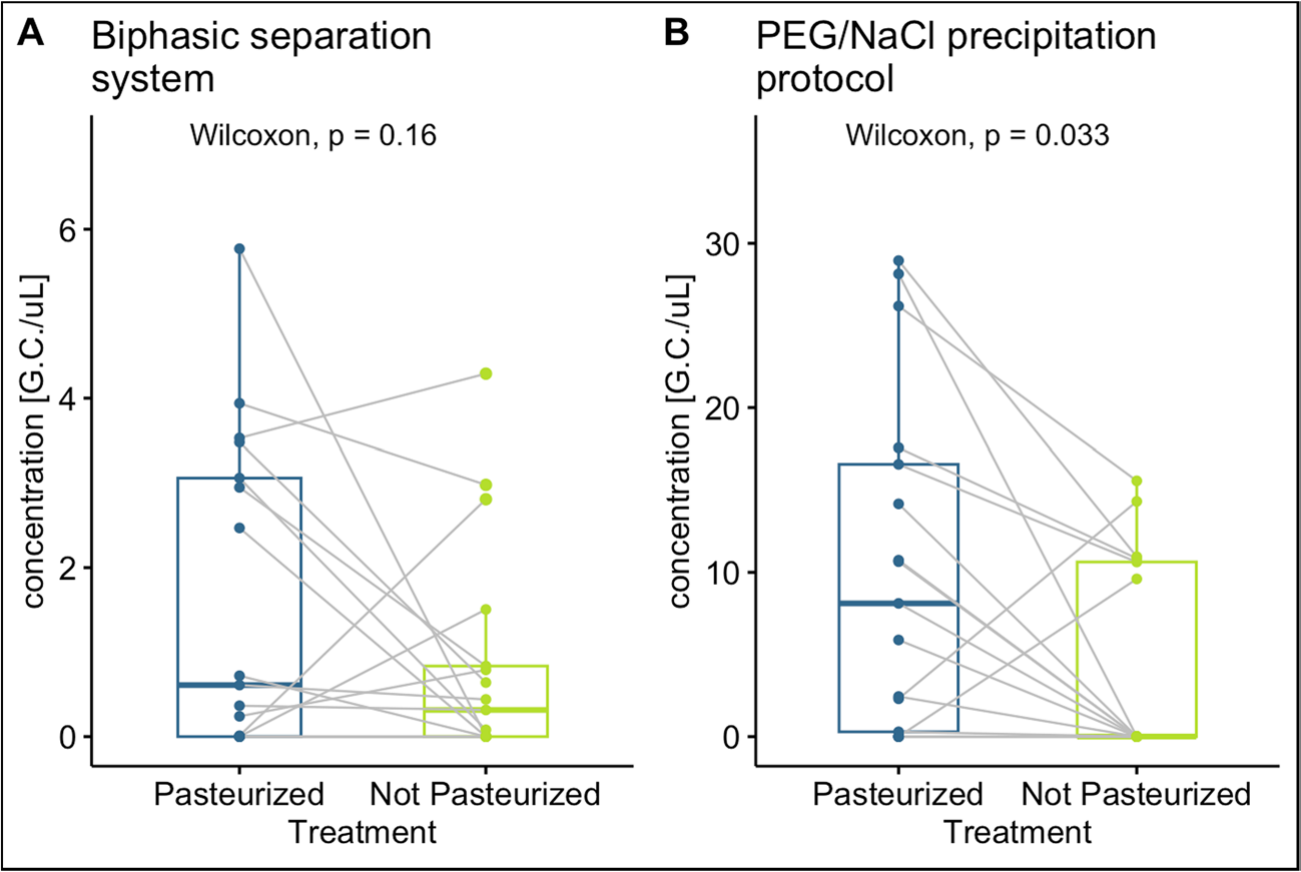
SARS-CoV-2 concentrations comparing pasteurized and non-pasteurized samples concentrated with **A** biphasic separation system and **B** PEG/NaCl precipitation protocol

Figure 7 shows the results obtained from pasteurized and not pasteurized samples for the two concentration methods. In Fig. 7A, the results of the biphasic separation method, evaluated with the Wilcoxon paired test, do not give statistically significant results. Instead, for the PEG/NaCl precipitation protocol, the concentrations in the not pasteurized samples resulted in slightly lower concentrations, with a weak statistical significance. The results confirm the usefulness of applying the pasteurization pre-treatment as it enhances safety for the operators and maintains the identification of the viral titer. In addition, according to the literature, pasteurization increases the repeatability of sample quantification (Hemati et al. 2021; Trujillo et al. 2021). The discrepancies between our results and other publications in the literature, which have found that heat pre-treatment is detrimental to the recovery of viral titer (Islam et al. 2022), may be due to different pasteurization temperatures and times, as indicated by Whitney et al. (2021).

## Conclusions

In conclusion, based on the results obtained, the PEG/NaCl precipitation method is more efficient and preferable over the biphasic separation system for the concentration of SARS-CoV-2 in wastewater. The disadvantage of the PEG/NaCl precipitation method is the startup costs in case a suitable centrifuge is unavailable (LaTurner et al. 2021). However, this disadvantage is balanced by the efficiency of the analysis even at low initial concentrations of the virus, the possibility of analyzing multiple samples at the same time, and faster analysis time, which is approximately 4 h compared to the 12 and more hours of the biphasic separation analysis. Several issues are associated with the biphasic protocol: the need for specific manual skills with separating funnels, leaving the funnels unattended overnight, and, above all, the use of chloroform, a carcinogenic substance. This can lead to the generation of a larger volume of hazardous waste, which must be disposed properly. So, the results of this study show that the PEG/NaCl precipitation is a more efficient and reliable method for the concentration of SARS-CoV-2 in wastewater compared to the biphasic separation system. Additionally, pasteurization as pre-treatment and storage of samples at 4.3±0.2°C are recommended to ensure the safety of operators and maintain viral titres. These data will support the international standardization of methods for the detection of SARS-CoV-2 in wastewater, which will help to compare results across different regions and countries.

### Supplementary information

ESM 1 (DOCX 111 kb)

## Data Availability

The data that supports the findings of this study are available within the article and its supplementary material. All the materials used were cited within the paper.

## References

[CR1] Ahmed W, Angel N, Edson J, Bibby K, Bivins A, O’Brien JW, Choi PM, Kitajima M, Simpson SL, Li J, Tscharke B, Verhagen R, WJM S, Zaugg J, Dierens L, Hugenholtz P, Thomas KV, Mueller JF (2020). First confirmed detection of SARS-CoV-2 in untreated wastewater in Australia: a proof of concept for the wastewater surveillance of COVID-19 in the community. Sci Total Environ.

[CR2] Cutrupi F, Cadonna M, Manara S, Postinghel M, La Rosa G, Suffredini E, Foladori P (2022). The wave of the SARS-CoV-2 Omicron variant resulted in a rapid spike and decline as highlighted by municipal wastewater surveillance. Environ Technol Innov.

[CR3] Dimitrakopoulos L, Kontou A, Strati A, Galani A, Kostakis M, Kapes V, Lianidou E, Thomaidis N, Markou A (2022). Evaluation of viral concentration and extraction methods for SARS-CoV-2 recovery from wastewater using droplet digital and quantitative RT-PCR. Case Stud Chem Environ Eng.

[CR4] ggplot2 (2016) https://ggplot2.tidyverse.org/. Accessed 2.13.23x

[CR5] He X, EHY L, Wu P, Deng X, Wang J, Hao X, Lau YC, Wong JY, Guan Y, Tan X, Mo X, Chen Y, Liao B, Chen W, Hu F, Zhang Q, Zhong M, Wu Y, Zhao L, Zhang F, Cowling BJ, Li F, Leung GM (2020). Temporal dynamics in viral shedding and transmissibility of COVID-19. Nat Med.

[CR6] Hemati M, Soosanabadi M, Ghorashi T, Ghaffari H, Vahedi A, Sabbaghian E, Rasouli Nejad Z, Salati A, Danaei N, Kokhaei P (2021). Thermal inactivation of COVID-19 specimens improves RNA quality and quantity. J Cell Physiol.

[CR7] Huang Y, Zhou N, Zhang S, Yi Y, Han Y, Liu M, Han Y, Shi N, Yang L, Wang Q, Cui T, Jin H (2022). Norovirus detection in wastewater and its correlation with human gastroenteritis: a systematic review and meta-analysis. Environ Sci Pollut Res Int.

[CR8] Islam G, Gedge A, Lara-Jacobo L, Kirkwood A, Simmons D, Desaulniers J-P (2022). Pasteurization, storage conditions and viral concentration methods influence RT-qPCR detection of SARS-CoV-2 RNA in wastewater. Sci Total Environ.

[CR9] Jiang G, Liu Y, Tang S, Kitajima M, Haramoto E, Arora S, Choi PM, Jackson G, D'Aoust PM, Delatolla R, Zhang S, Guo Y, Wu J, Chen Y, Sharma E, Prosun TA, Zhao J, Kumar M, Honda R, Ahmed W, Meiman J (2023). Moving forward with COVID-19: future research prospects of wastewater-based epidemiology methodologies and applications. Curr Opin Environ Sci Health.

[CR10] Juel MAI, Stark N, Nicolosi B, Lontai J, Lambirth K, Schlueter J, Gibas C, Munir M (2021). Performance evaluation of virus concentration methods for implementing SARS-CoV-2 wastewater based epidemiology emphasizing quick data turnaround. Sci Total Environ.

[CR11] Kaya D, Niemeier D, Ahmed W, Kjellerup BV (2022). Evaluation of multiple analytical methods for SARS-CoV-2 surveillance in wastewater samples. Sci Total Environ.

[CR12] La Rosa G, Iaconelli M, Mancini P, Bonanno Ferraro G, Veneri C, Bonadonna L, Lucentini L, Suffredini E (2020). First detection of SARS-CoV-2 in untreated wastewaters in Italy. Sci Total Environ.

[CR13] La Rosa G, Mancini P, Bonanno Ferraro G, Veneri C, Iaconelli M, Bonadonna L, Lucentini L, Suffredini E (2021). SARS-CoV-2 has been circulating in northern Italy since December 2019: Evidence from environmental monitoring. Sci Total Environ.

[CR14] LaTurner ZW, Zong DM, Kalvapalle P, Gamas KR, Terwilliger A, Crosby T, Ali P, Avadhanula V, Santos HH, Weesner K, Hopkins L, Piedra PA, Maresso AW, Stadler LB (2021). Evaluating recovery, cost, and throughput of different concentration methods for SARS-CoV-2 wastewater-based epidemiology. Water Res.

[CR15] Lu X, Wang L, Sakthivel SK, Whitaker B, Murray J, Kamili S, Lynch B, Malapati L, Burke SA, Harcourt J, Tamin A, Thornburg NJ, Villanueva JM, Lindstrom S (2020) US CDC real-time reverse transcription PCR panel for detection of severe acute respiratory syndrome coronavirus 2. Emerg Infect Dis 26. 10.3201/eid2608.201246 10.3201/eid2608.201246 PMC7392423 32396505

[CR16] Mallapaty S (2020). How sewage could reveal true scale of coronavirus outbreak.

[CR17] Markt R, Mayr M, Peer E, Wagner AO, Lackner N, Insam H (2021) Detection and stability of SARS-CoV-2 fragments in wastewater: impact of storage temperature. Pathogens 10. 10.3390/pathogens10091215 10.3390/pathogens10091215 PMC8471725 34578246

[CR18] Melvin RG, Chaudhry N, Georgewill O, Freese R, Simmons GE (2021) Predictive power of SARS-CoV-2 wastewater surveillance for diverse populations across a large geographical range. medRxiv. 10.1101/2021.01.23.21250376

[CR19] Ministero della Salute (2020) ISS, al via la rete “sentinella” di sorveglianza epidemiologica del coronavirus nelle acque reflue. https://www.salute.gov.it/portale/nuovocoronavirus/dettaglioNotizieNuovoCoronavirus.jsp?lingua=italiano&menu=notizie&p=dalministero&id=4953. Accessed 13 Feb 2023

[CR20] Nordmann E, McAleer P, Toivo W, Paterson H, LM DB (2022). Data visualization using R for researchers who do not use R. Adv Methods Pract Psychol Sci.

[CR21] Oloye FF, Xie Y, Challis JK, Femi-Oloye OP, Brinkmann M, McPhedran KN, Jones PD, Servos MR, Giesy JP (2023). Understanding common population markers for SARS-CoV-2 RNA normalization in wastewater - a review. Chemosphere.

[CR22] Pecson BM, Darby E, Haas CN, Amha YM, Bartolo M, Danielson R, Dearborn Y, Di Giovanni G, Ferguson C, Fevig S, Gaddis E, Gray D, Lukasik G, Mull B, Olivas L, Olivieri A, Qu Y, SARS-CoV-2 Interlaboratory Consortium (2021). Reproducibility and sensitivity of 36 methods to quantify the SARS-CoV-2 genetic signal in raw wastewater: findings from an interlaboratory methods evaluation in the U.S. Environ Sci.

[CR23] Philo SE, Keim EK, Swanstrom R, Ong AQW, Burnor EA, Kossik AL, Harrison JC, Demeke BA, Zhou NA, Beck NK, Shirai JH, Meschke JS (2021). A comparison of SARS-CoV-2 wastewater concentration methods for environmental surveillance. Sci Total Environ.

[CR24] Randazzo W, Truchado P, Cuevas-Ferrando E, Simón P, Allende A, Sánchez G (2020). SARS-CoV-2 RNA in wastewater anticipated COVID-19 occurrence in a low prevalence area. Water Res.

[CR25] Röder B, Frühwirth K, Vogl C, Wagner M, Rossmanith P (2010). Impact of long-term storage on stability of standard DNA for nucleic acid-based methods. J Clin Microbiol.

[CR26] The R Project for Statistical Computing (2021) https://www.r-project.org/. Accessed 2.13.23

[CR27] Trujillo M, Cheung K, Gao A, Hoxie I, Kannoly S, Kubota N, San KM, Smyth DS, Dennehy JJ (2021). Protocol for safe, affordable, and reproducible isolation and quantitation of SARS-CoV-2 RNA from wastewater. PLoS One.

[CR28] Wang W, Xu Y, Gao R, Lu R, Han K, Wu G, Tan W (2020). Detection of SARS-CoV-2 in different types of clinical specimens. JAMA.

[CR29] Whitney ON, Kennedy LC, Fan VB, Hinkle A, Kantor R, Greenwald H, Crits-Christoph A, Al-Shayeb B, Chaplin M, Maurer AC, Tjian R, Nelson KL (2021). Sewage, salt, silica, and SARS-CoV-2 (4S): an economical kit-free method for direct capture of SARS-CoV-2 RNA from wastewater. Environ Sci Technol.

[CR30] WHO (2003) Guidelines for environmental surveillance of poliovirus circulation (No. WHO/V&B/03.03).2-TP-03.03 (who.int). Accessed 2.13.23

[CR31] WHO (2020) WHO Director-General’s opening remarks at the media briefing on COVID-19 - 11 March 2020. https://www.who.int/director-general/speeches/detail/who-director-general-s-opening-remarks-at-the-media-briefing-on-covid-19%2D%2D-11-march-2020. Accessed 2.13.23

[CR32] WHO (2023a) Statement on the fifteenth meeting of the IHR (2005) Emergency Committee on the COVID-19 pandemic. https://www.who.int/news/item/05-05-2023-statement-on-the-fifteenth-meeting-of-the-international-health-regulations-(2005)-emergency-committee-regarding-the-coronavirus-disease-(covid-19)-pandemic. Accessed 8.1.23

[CR33] WHO (2023b) From emergency response to long-term COVID-19 disease management: sustaining gains made during the COVID-19 pandemic. https://www.who.int/publications/i/item/WHO-WHE-SPP-2023.1. Accessed 8.1.23

[CR34] Wu F, Zhang J, Xiao A, Gu X, Lee WL, Armas F, Kauffman K, Hanage W, Matus M, Ghaeli N, Endo N, Duvallet C, Poyet M, Moniz K, Washburne AD, Erickson TB, Chai PR, Thompson J, Alm EJ (2020) SARS-CoV-2 titers in wastewater are higher than expected from clinically confirmed cases. mSystems 5. 10.1128/mSystems.00614-20 10.1128/mSystems.00614-20 PMC7566278 32694130

[CR35] Zuckerman NS, Bar-Or I, Sofer D, Bucris E, Morad H, Shulman LM, Levi N, Weiss L, Aguvaev I, Cohen Z, Kestin K, Vasserman R, Elul M, Fratty IS, Geva M, Wax M, Erster O, Yishai R, Hecht-Sagie L et al (2022) Emergence of genetically linked vaccine-originated poliovirus type 2 in the absence of oral polio vaccine, Jerusalem, April to July 2022. Euro Surveill 27. 10.2807/1560-7917.ES.2022.27.37.2200694 10.2807/1560-7917.ES.2022.27.37.2200694 PMC9479469 36111556

