# Supplementary material for: Evaluation of concentration procedures, sample pre-treatment, and storage condition for the detection of SARS-CoV-2 in wastewater
Source: Environ Sci Pollut Res Int. 2023 Sep 21;30(48):106660–70. doi: 10.1007/s11356-023-29696-y (PMC10579110; doi:10.1007/s11356-023-29696-y)
Supplement: Supplementary file 1 — (DOCX 111 kb) [file 11356_2023_29696_MOESM1_ESM.docx]

**SUPPLEMENTARY MATERIAL**

**Assessment of recovery of the concentration methods**

| Primer name | Nucleotide sequence (5’-3’) | References |
| --- | --- | --- |
| Mengo 110 (FW) | GCG GGT CCT GCC GAA AGT |  |
| Mengo 209 (REV) | GAA GTA ACA TAT AGA CAG ACG CAC AC | Pintò et al., 2009 |
| Mengo 147 (PROBE) | FAM-ATC ACA TTA CTG GCC GAA GC-MGBNFQ |  |

*Table 2. Primers and probe for the determination of Mengovirus (MgV) process control virus.*

**Additional testing performed by the Region Liguria using the N1 as a target gene**

Additional comparative testing of the two concentration methods (biphasic separation system and PEG/NaCl precipitation protocol) was performed on 24 samples using, as detection method a real-time RT-qPCR targeting the N1 gene (Lu et al., 2020). Primers are shown in Table 3.

| Primer name | Nucleotide sequence (5’-3’) | Genome Location | Reference |
| --- | --- | --- | --- |
| 2019-nCoV_N1-F | GACCCCAAAATCAGCGAAAT | 28287 - 28306 | [Lu et al. 2020](https://paperpile.com/c/e3AZEZ/TIbZ) |
| 2019-nCoV_N1-R | TCTGGTTACTGCCAGTTGAATCTG | 28335 - 28358 |  |
| 2019-nCoV_N1-P | FAM-ACCCCGCATTACGTTTGGTGGACC-BHQ1 | 28309 - 28332 |  |

*Table 3. Primers and probes used in the study; Nucleotide numbering based on SARS-CoV-2 (accession no. NC_045512).*

The N1 RT-qPCR assays were performed using the AgPath-ID™ (Applied Biosystem-ThermoFisher) one-step RT-PCR reagent kit. Each 20 μL reaction contained 125 nM of 2019-nCoV_N1-P, 500 nM of both 2019-nCoV_N1-F and 2019-nCoV_N1-R primers, 8.5 μL of nuclease-free water and 5 μl of nucleic acid extract. The thermocycling conditions consisted of 15 min at 50°C for reverse transcription, 2 min at 95°C for the activation of the Taq enzyme, and 45 cycles of 3 s at 95°C and 30 s at 55°C. The dsDNA N1 standard was provided by the TIB Molbiol Srl (Genova, Italy).


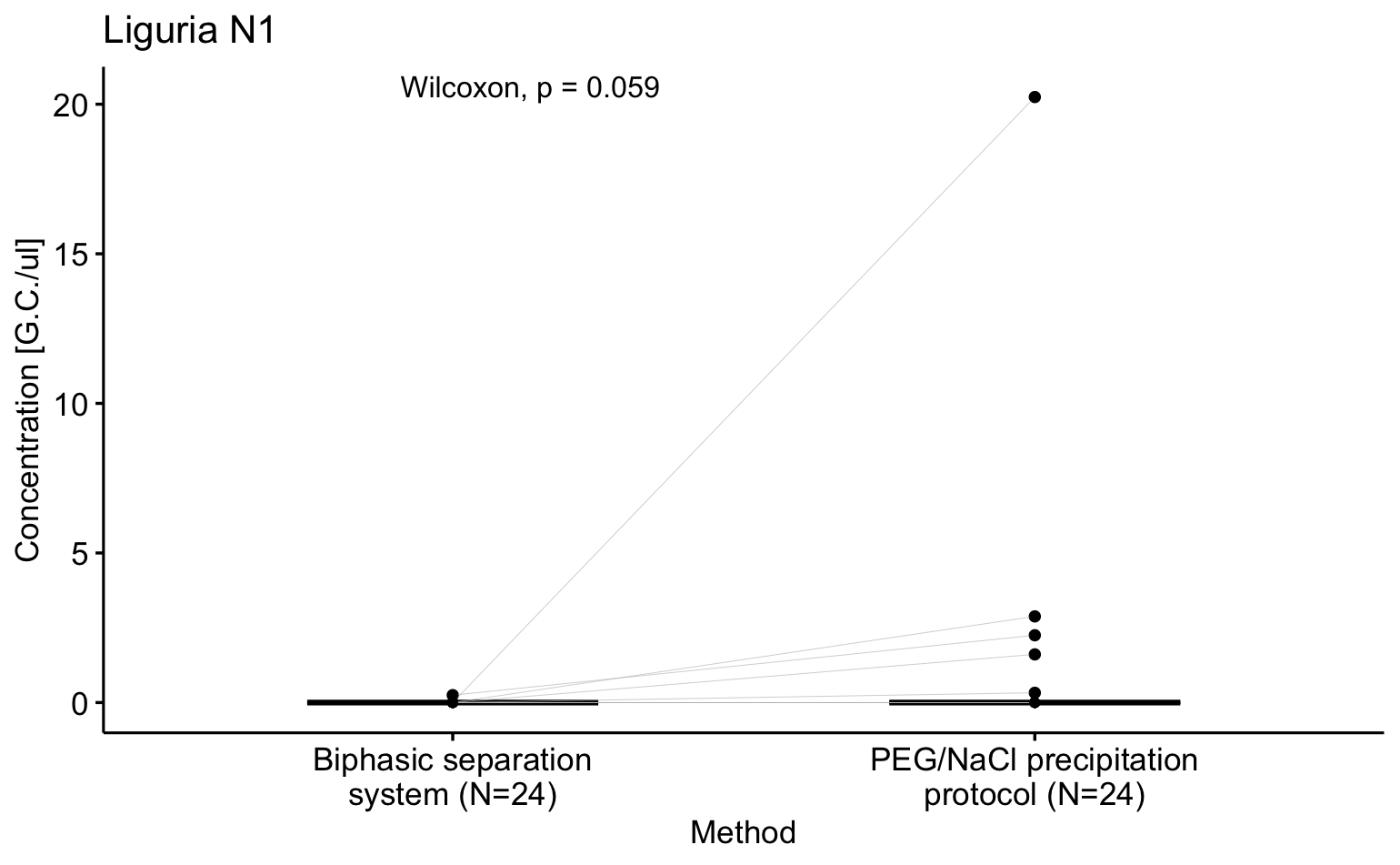


*Supplementary Fig.7. Comparison of SARS-CoV-2 concentration measured for each method (biphasic separation system and PEG/NaCl precipitation protocol)of Liguria samples for molecular target N1*

Only one sample tested positive for SARS-CoV-2 using the biphasic method, while 5 samples tested positive with the PEG/NaCl method. In this case, the Wilcoxon paired test did not show a significant difference between the two methods; however, the higher number of positive results obtained with the PEG/NaC, searching for the ORF-1b target, confirmed the reliability of this concentration approach.
